# RABEX-5 plays an oncogenic role in breast cancer by activating MMP-9 pathway

**DOI:** 10.1186/1756-9966-32-52

**Published:** 2013-08-13

**Authors:** Xiang Zhang, Jie Min, Yingjian Wang, Yan Li, Hongzhong Li, Qiang Liu, Xinjie Liang, Peng Mu, Hongyuan Li

**Affiliations:** 1 Molecular Oncology and Epigenetics Laboratory, The First Affiliated Hospital of Chongqing Medical University, Chongqing 400016, China; 2 Department of Endocrine and breast Surgery, The First Affiliated Hospital of Chongqing Medical University, Chongqing 400016, China

**Keywords:** RABEX-5, Breast cancer, Oncogene, RNAi

## Abstract

**Background:**

RABEX-5, a guanine nucleotide exchange factor (GEF) for RAB-5, plays an important role in cell mobility and altered expression associated with tumor metastasis. This study aimed to investigate the role of RABEX-5 in proliferation and metastasis of breast cancer in vitro and ex vivo.

**Methods:**

RABEX-5 expression was examined in breast cancer, benign tumor and normal breast tissues by immunohistochemistry and western blot. Two stable cell lines were established, the MCF-7/NC negative control cell line and the MCF-7/KD cell line, which stably expressed an RNA interference (RNAi) construct that induced downregulation of RABEX-5 expression. These cell lines were utilized to evaluate the role of RABEX-5 in cell proliferation and migration in vitro and tumorigenicity in vivo. The possible role of RABEX-5 in the regulation of matrix metallopeptidase 9 (MMP-9) was evaluated using western blot and real-time PCR.

**Results:**

RABEX-5 expression was found to be significantly higher in breast cancer tissues compared with benign tumor and normal breast tissues. High levels of RABEX-5 expression were associated with axillary lymph node metastasis. In addition, RABEX-5 silencing significantly reduced cancer cell proliferation, colony formation and migration ability in vitro and inhibited tumor growth in vivo. RABEX −5 knockdown also attenuated the migration of breast cancer cells via modulation of MMP-9 transcriptional activity.

**Conclusions:**

Our results indicate that RABEX-5 plays an oncogenic role in breast cancer by modulating the proliferation and metastasis potential of breast cancer cells. Thus, RABEX-5 is a promising prognostic indicator for patients with breast cancer.

## Introduction

Breast cancer is a systemic disease. It has become the most frequently diagnosed cancer and the leading cause of cancer death in females worldwide, with rapidly increasing incidence and mortality rates. Breast cancer accounted for 23% (1.38 million) of total new cancer cases and 14% (458,400) of total cancer deaths in 2008 [1]. The incidence rates of breast cancer vary from 19.3 per 100,000 women in Eastern Africa to 89.7 per 100,000 women in Western Europe, while the mortality rate is approximately 6–19 per 100,000 [2].

Tumorigenesis is a multifactor, multistep complex process that involves the cooperation of many genes, in particular the activation of oncogenes and inactivation of tumor suppressor genes. Recent clinical data have emerged demonstrating that Ras family genes play important roles in human tumorigenesis. The activation of Ras proteins by mutational activation or by growth factor stimulation is a common occurrence in many human cancers and was shown to induce and to be required for tumor growth. The Ras superfamily of small guanosine triphosphatases (GTPases) contains over 150 human members, with the Ras oncogene proteins as the founding members of this family, which is divided into five major branches on the basis of sequence and functional similarities: Ras, Rho, Rab, Ran and Arf.

Small GTPases share a common biochemical mechanism. The Ras superfamily of GTPases function as GDP/GTP-regulated molecular switches. They alternate between GTP- and GDP-bound conformations in which the GTP-bound conformation represents the “on” state and the GDP-bound conformation represents the “off” state. Upon binding, two regions of Ras undergo dramatic structural changes depending on the type of bound nucleotide [3]. Small GTPases exhibit high-affinity binding for GDP and GTP and possess low intrinsic GTP hydrolysis and GDP/GTP exchange activities. GDP/GTP cycling is controlled by two main classes of regulatory proteins. Guanine-nucleotide-exchange factors (GEFs) promote the formation of the active, GTP-bound form, whereas GTPase-activating proteins (GAPs) accelerate the intrinsic GTPase activity to promote formation of the inactive, GDP-bound form [4,5]. GTPases within a branch use shared and distinct GAPs and GEFs. GTPases in different branches exhibit structurally distinct but mechanistically similar GAPs and GEFs. The two nucleotide-bound states have similar conformations but have pronounced differences corresponding to the switch I (Ras residues 30–38) and switch II (59–67) regions; the GTP-bound conformation possesses high affinity for effector targets [6,7]. It is mainly through the conformational changes in these two switches that the regulatory proteins and effectors modulate the nucleotide status of the small GTPases [8].

Ras-associated binding (Rab)-GTPases are members of the Ras family of small GTPases. The Rab family proteins regulate a variety of membrane trafficking events in all eukaryotic cells by recruiting specific effector molecules. They are important for the regulation of signal transduction and cellular processes such as differentiation, proliferation, vesicle transport, nuclear assembly and cytoskeleton formation, and they are abnormally expressed in various cancer tissues [9]. Rab GTPases regulate membrane trafficking between organelles by recruitment of effector proteins. Immunodeficiencies, cancer, and neurological disorders are associated with functional impairments of the Rab signaling pathways [10]. Alterations or mutations in the Rab proteins and their effectors have been suggested to cause many human diseases, including cancer. In particular, previous reports have demonstrated that alterations in RAB-25, RAB-7, RAB-5, and RAB-11 could cause different types of cancer. Rab family proteins are also involved in exocytosis in endocrine cells and are associated with the invasive and metastatic potential of breast cancer by promoting the production of insulin-like growth factor-II (IGF-II). The rate of secretion controls the expression of vascular endothelial growth factor (VEGF), matrix metalloproteinase-9 (MMP-9), cathepsin D, cyclin D1, p16, and urokinase-type plasminogen activator [11].

The small GTPase RAB-5, which is found at the plasma membrane and early endosomes, is a master regulator of early endocytic trafficking [12]. Like other small GTPases, RAB-5 is activated by an exchange of bound GDP with GTP, which is catalyzed by a family of guanine-nucleotide-exchange factors (GEFs). RABEX-5 was identified as an interactor of Rabaptin-5 and was found to possess GEF activity toward RAB-5 and related GTPases. Likewise, both Rabaptin-5 and RABEX-5 are essential for RAB-5-driven endosome fusion in vitro [13].

Aberrant RABEX-5 expression may result in obstruction of the RAB-5-mediated endocytic vesicle fusion process, thereby causing defects in phagocytosis. A previous study found that RABEX-5 was over-expressed in colorectal tumor cell lines [14]. The authors also hinted that RABEX-5 may act as an oncogene that is involved in the formation and development of malignant tumors and might influence tumor biological behavior. However, the role and mechanism of action of RABEX-5 in breast cancer carcinogenesis and progression have not yet been determined. In this study, we first analyzed the expression of RABEX-5 in breast cancer tissue and breast cancer cell lines by immunohistochemistry and real-time PCR. Subsequently, the influence of the biological function of breast cancer was evaluated in vitro and vivo. Our results showed that RABEX-5 was overexpressed and plays a distinct oncogenic role in breast cancer.

## Materials and methods

### Patients and tissue samples

A total of 60 invasive ductal carcinoma breast tissue samples, 15 normal breast tissue samples, and 15 benign breast tumor tissue samples were obtained from patients who underwent surgical treatment at the First Affiliated Hospital of Chongqing Medical University from February to May in 2009 after obtaining informed consent. None of the patients received therapy before surgery. The tissues from all of the patients were staged according to the American Joint Committee on Cancer (AJCC) breast cancer TNM staging system: stage I, n = 29; stage II, n = 25; and stage III, n = 6. All tissue samples were fixed in 10% formalin and then embedded in paraffin for histologic examination.

### Immunohistochemistry

Immunohistochemical staining was performed on paraffin-embedded specimens. Slides were routinely deparaffinized and hydrated. Endogenous peroxidase was blocked with 3% hydrogen peroxide for 10 min, and the deparaffinized sections in 10 mM citrate buffer were microwaved for 30 minutes for epitope retrieval. Then, the sections were incubated with an antibody against RABEX-5 (1:50 dilution, Santa Cruz Biotechnology, USA) and an antibody against MMP-9 (1:100 dilution, Ab76003, Abcam, UK) for 18 h at 4°C in 2% bovine serum albumin in Phosphate-buffered saline (PBS). A secondary antibody was added and incubated for 1 h at 37°C. The sections were counterstained with hematoxylin for 3–5 min. PBS, instead of primary antibody, was used as a negative control. For the evaluation of expression, IPP (version 6.0, Media Cybernetics, Silver Spring, MD) was used as described previously [15]. Briefly, 5 digital images at 1360×1024 pixel resolution and 400 × magnification were captured by the LEICA DM500 ICC50 microscope (Leica Microsystems, Germany). The measurement parameters included area, sum, and IOD, and the values were counted.

### Cell lines and culture conditions

Five breast cancer cell lines (MCF-7, MDA-MB-231, BT549, T47D and SKBR3) were used. All cell lines were obtained from the Molecular Oncology and Epigenetics Laboratory of The First Affiliated Hospital of Chongqing Medical University. Cell lines were routinely maintained in RPMI 1640 medium supplemented with 10% fetal bovine serum (GIBCO, Grand Island, NY) in a 5% CO_2_ atmosphere at 37°C.

### RNA extraction, reverse transcription, and real-time PCR analysis

Total RNA was isolated from tissues and cells using Trizol (Invitrogen, USA) according to the manufacturer’s instructions. Reverse transcription was performed using random hexamers, and reverse transcription-PCR using Go-Taq (Promega, Madison, WI, USA), with GAPDH as a control, was performed using the following primers: RABEX-5 F: 5′-TTGGACAGATGGAATTGCAA-3′ and RABEX-5R: 5′-GTTGCAGTGGTGGAGGAAGT-3′. The PCR program consisted of initial denaturation at 95°C for 2 min, followed by 32 cycles (for RABEX-5) or 23 cycles (for GAPDH) of the reaction (94°C for 30 s, 55°C for 30 s and 72°C for 30 s), with a final extension at 72°C for 10 min. Quantitative real-time PCR was performed using the SYBR Premix Ex Taq™ kit (TAKARA, Japan). After an initial denaturation step at 95°C for 30 s, thermal cycling was initiated. Each cycle consisted of 95°C for 5 s and 60°C for 34 s. The fluorescent signal was acquired at the end of the elongation step. A total of 40 cycles was performed. The nucleotide sequences of the primers used for PCR amplification were as follows: (1)RABEX-5 F: 5′-TTGGACAGATGGAATTGCAA-3′ and RABEX-5R: 5′- GTTGCAGTGGTGGAGGAAGT-3′(GenBank accession No. NM_004994), (2)MMP-9 F: 5′-CCTGGAGACCTGAGAACCAATC-3′ and MMP-9R: 5′-CCACCCGAGTGTAACCATAGC-3′(GenBank accession No. NM_014504), (3)GAPDH-F: 5′-TCCTGTGGCATCCACGAAACT-3′ and GAPDH-R: 5′-GAAGCATTTGCGGTGGACGAT-3′(GenBank accession No. NM_001101). The comparative Ct (threshold cycle) method was used to calculate the relative changes in gene expression obtained from the real-time PCR system.

### RNA interference

An siRNA vector was generated by ligating DNA oligos into the linear pMAGic-siR lentiviral plasmid vector. This vector was used to inhibit human RABEX-5 gene expression (GenBank accession No. NM_014504). As a control, the pMAGic-siR-neg lentiviral control plasmid encoding an mRNA not known to target any vertebrate gene was used. The RABEX-5 siRNA targeting oligo was 5′-GGATGCAAACTCGTGGGAA-3′, while the non-homologous sequence used as the control was 5′- TTCTCCGAACGTGTCACGT-3′.

After the lentiviral vector to perform RNA interference (RNAi) of the RABEX-5 gene was constructed, the recombinant lentiviral plasmid and the control lentiviral plasmid were transfected into MCF-7 cells. The cells with the most appropriate level of transfection were selected. Real-time PCR and western blot analyses were used to examine the expression of RABEX-5.

### Colony formation assay and cell proliferation assay

MCF-7 cells transfected with the pMAGic-siR lentiviral plasmid vector (MCF-7/KD) and the pMAGic-siR-neg lentiviral control plasmid (MCF-7/NC) were plated in 6-well plates (2×10^3^ cells/well). The number of colonies (>50 cells per colony) was counted after staining with Giemsa 14 days later, and the colonies were photographed. Each experiment was performed in triplicate three times.

A Cell Count Kit-8 (CCK-8, Beyotime, China) was employed to quantitatively evaluate cell viability. Briefly, 2×10^3^ cells/well were seeded in 96-well flat-bottomed plates, then grown at 37°C for, 24, 48, 72, and 96 h. Then, the original medium in each well was replaced by 200 μl 10% FBS/RPMI 1640 medium contain 20 μl CCK-8. The cells were incubated at 37°C for 2 h, and the absorbance was determined at wavelengths of 450 nm and 630 nm (calibrated wave) using a microplate reader. RPMI 1640 containing 10% CCK-8 was used as a control.

### Wound healing assay and transwell cell migration assay

The mobility of MCF-7/KD and MCF-7/NC cells was assessed using a scratch wound assay. We drew horizontal lines across the back of the wells of 6-well plates with a marker pen. The cells (5×10^5^ cells/well) were plated into the 6-well plates. On the following day, the confluent cell monolayers were carefully wounded (perpendicular to the horizontal lines) with sterile pipette tips and washed with PBS twice to remove cellular debris. Serum-free medium was added into the wells. Then, the wounded cell monolayers were cultured for another 48 h. Images were captured under a fluorescent microscope to observe the distribution of the cells at the scratch zone at different timepoints.

A cell migration assay was performed using transwell chambers with a pore size of 0.8 μm. A total of 1×10^5^ cells were seeded in serum-free medium in the upper chamber, while medium containing 10% FBS was added as a chemoattractant to the lower chamber. After incubating for 48 h at 37°C, the cells in the upper chamber were carefully removed with a cotton swab, and the cells that had migrated to the reverse face of the membrane were fixed in methanol, stained with Giemsa, and counted.

### Mouse tumor transplantation models

In vivo studies were conducted in immunodeficient mice. Six female athymic mice, weighing 18–20 g at 4 weeks of age, were obtained from the Beijing Laboratory Animal Research Center (Beijing, China). All mice were handled according to the recommendations of the National Institutes of Health Guidelines for Care and Use of Laboratory Animals. The MCF-7/KD cells were inoculated subcutaneously into the right flanks of the mice (2×10^6^ cells/mouse), while the MCF-7/NC cells were inoculated subcutaneously into the left flanks of the mice (2×10^6^ cells/ mouse). Tumor size was measured externally every 3 days using a caliper, and tumor volume was estimated using the equation: length (mm) × width^2^ (mm) × 0.52. The mice were sacrificed 4 weeks after the transplant, and the tumors were weighed after dissection. Samples from each area were snap-frozen at −80°C for protein preparation, and the corresponding tissue samples were fixed in 4% formalin to obtain paraffin-embedded sections.

### Western blot

After protein lysates were prepared, an equivalent amount of protein from each sample was loaded onto an SDS polyacrylamide gel. The protein lysates were then separated by SDS-PAGE and electroblotted onto PVDF membranes. After blocking with 5% nonfat milk and 0.1% Tween 20 in TBS, the membranes were incubated with rabbit anti-RABEX-5 (1:200 dilution; Santa Cruz Biotechnology, USA), rabbit anti-MMP-9 (1:1000 dilution; Ab76003, Abcam, UK) and rabbit anti-GAPDH (1:2000 dilution; Ab9485, Abcam, UK) antibodies overnight at 4°C. Then, the membranes were incubated with a horseradish peroxidase-conjugated secondary antibody at a dilution of 1:1000 for 1 h. The blots were visualized using ECL Plus Western Blotting Detection Reagents (Beyotime, China) and scanned.

### Statistical analyses

Statistical analyses were performed using SPSS 16.0 software. Expression analysis, original real-time PCR data, western blot data, migration data, and colony formation data were recorded as continuous variables and analyzed using Student’s t-test. Differences were considered statistically significant if the P value was less than 0.05.

## Results

### Expression of RABEX-5 in tissues and breast cancer cell lines

**Figure 1:**
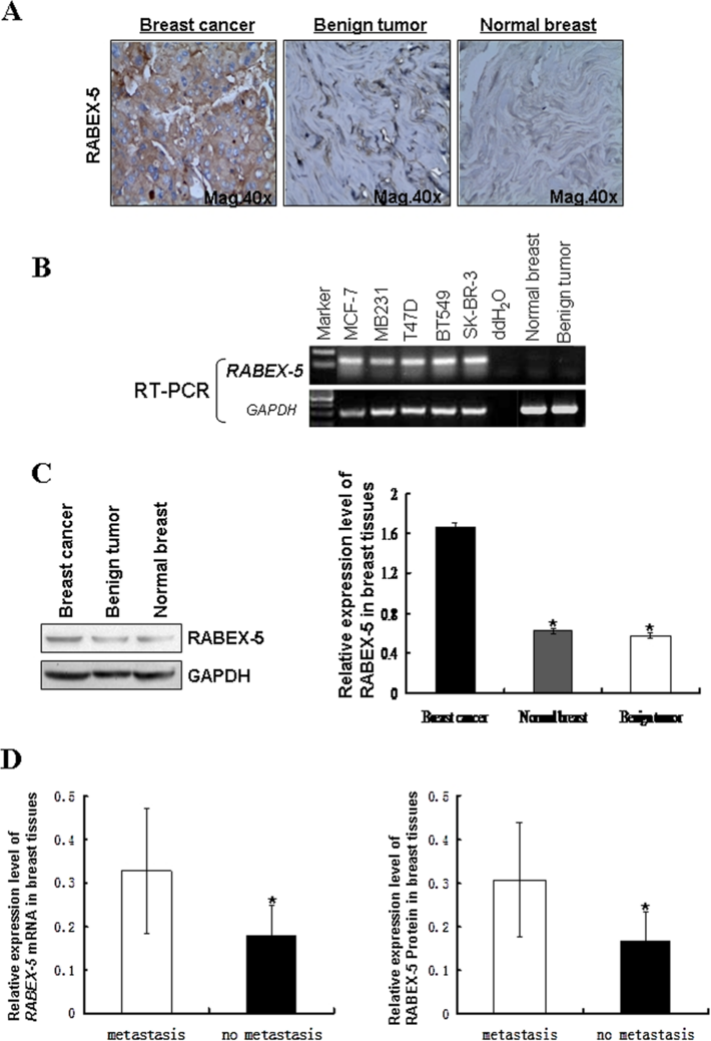
**Expression of RABEX-5 in breast cancer. (A)**, Expression of RABEX-5 in Breast cancer, Benign tumor, and Normal breast tissue. The distinct brown staining was located in the cytoplasm of positive cells. **(B)**, Benign tumor tissue, Normal breast tissue and breast cancer cell lines were evaluated using semi-quantitative RT-PCR, with GAPDH as a control. **(C)**, RABEX-5 protein expression was detected in breast cancer tissue, Benign tumor tissue and Normal breast tissue by western blot. **(D)**, Expression of RABEX-5 and its relationship with axillary lymph node metastases.

We first examined the expression of RABEX-5 using IHC in breast cancer, benign breast tumor and normal breast tissues. The immunostaining quantification of RABEX-5 was analyzed using Image Pro-Plus (IPP). Our results showed that the RABEX-5 expression in breast cancer tissues was significantly higher than that in the benign breast tumor tissues and normal breast tissues (Figure 1A). Western blot analyses confirmed that RABEX-5 expression at the protein level was consistent with the IHC results (Figure 1C). Next, the expression level of RABEX-5 was analyzed in 5 breast cancer cell lines (MCF-7, MDA-MB-231, BT549, T47D, and SKBR3). RABEX-5 was overexpressed in all of the breast cancer cell lines (Figure 1B). These results suggest that RABEX-5 is frequently upregulated in breast cancer.

We further investigated the role of RABEX-5 in breast cancer by examining the relationship between RABEX-5 expression and the clinicopathologic features of breast cancer. RABEX-5 expression was associated with tumor size and axillary lymph node metastases (P<0.05) (Table 1, Figure 1D) but not with age, grade, and ER, PR, and C-erBb-2 status (P>0.05), suggesting that there is a relationship between RABEX-5 overexpression and breast cancer metastasis.

**Table 1:** Relationship of RABEX-5 mRNA and protein expression with clinicopathologic factors of breast cancer

**Group**	**NO.case**	**RABEX-5 mRNA level**	**RABEX-5 protein level**	**P value**
**Axillary lymph nodes**				P<0.001
**Metastasis**	27	0.329±0.144*	0.308±0.131*	
**No metastasis**	33	0.180±0.070*	0.168±0.066*	
**Tumor size(cm)**				P<0.05
**≤2 cm**	29	0.223±0.087	0.209±0.085	
**>2 cm,≤5 cm**	24	0.238±0.150#	0.222±0.140#	
**>5 cm**	7	0.358±0.139#	0.328±0.119#	
**Histologic grade**				P>0.05
**I**	29	0.229±0.138	0.205±0.128	
**II**	25	0.279±0.123	0.251±0.113	
**III**	6	0.299±0.127	0.279±0.123	
**ER**				P>0.05
**Positive**	27	0.276±0.159	0.256±0.145	
**Negative**	33	0.227±0.101	0.215±0.171	
**PR**				P>0.05
**Positive**	26	0.275±0.163	0.256±0.148	
**Negative**	34	0.228±0.099	0.216±0.097	
**HER-2**				P>0.05
**Positive**	16	0.232±0.128	0.217±0.119	
**Negative**	44	0.255±0.134	0.239±0.124	

# P<0.05, vs. tumor size >2 cm, ≤5 cm group and >5 cm group.

* P<0.001, vs. node metastasis group and no metastasis group.

### RABEX-5 gene downregulation in MCF-7 cells

**Figure 2:**
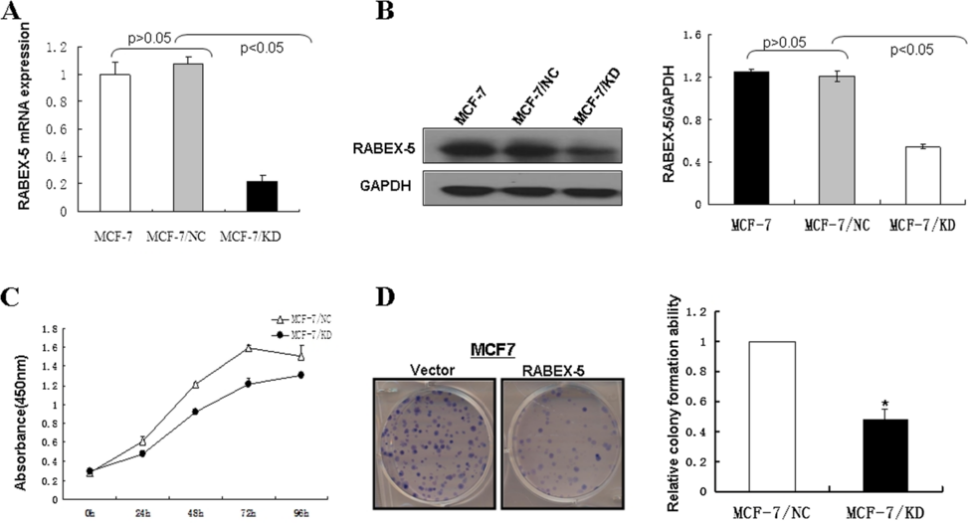
**Downregulation of RABEX-5 in MCF-7 cell and effects of RABEX-5 on the colony formation and cell proliferation of breast cancer cells. (A)**, RABEX-5 mRNA levels were analyzed by real time-PCR. MCF-7 cells were transfected with pMAGic-siR lentiviral plasmid (MCF-7/KD) and pMAGic-siR-neg lentiviral control plasmid (MCF-7/NC). **(B)**, RABEX-5 protein levels in MCF-7/KD and MCF-7/NC were analyzed by western blot. GAPDH was used as an internal control. P<0.05 compared with normal control (MCF-7) or MCF-7/NC. **(C)**, CCK-8 cell proliferation assay for vector- and RABEX-5-transfecetd MCF-7 cells, curves indicate a significant level of proliferation compared to controls(P <0.05). **(D)**, Representative colony formation assay, the numbers of colonies in MCF-7/NC were set to 100%. Values are expressed as mean±SD from three experiments, and the asterisks indicate statistical significance compared to controls (P<0.05).

To investigate whether decreased RABEX-5 expression can influence the biological behavior of breast cancer cell lines, an siRNA vector targeting the RABEX-5 gene was constructed. The effectiveness of the 4 siRNA oligos targeting RABEX-5 was verified (Table 2), and a reduction in the endogenous RABEX-5 expression was detected by real-time PCR in MCF-7 cells (P <0.05; data not shown). We selected the most promising candidate to be recombined into the RABEX-5-siRNA lentiviral vector, which was then transfected into MCF-7 cells (MCF-7/KD). MCF-7/KD cells showed a significant decrease in RABEX-5 mRNA and protein expression levels compared with MCF-7 cells (CON) or negative control-transduced cells (MCF-7/NC) (Figure 2A, Figure 2B).

**Table 2:** siRNA sequence-specific to RABEX-5

**Marker**	**Gene**	**Targetseq**
**pLVT540**	RABEX-5	CCCTCACATTCTCCAAGTT
**pLVT541**	RABEX-5	CCTTCCATAAACCGGCAAA
**pLVT542**	RABEX-5	GGATGCAAACTCGTGGGAA
**pLVT543**	RABEX-5	GCATCACCAAGTGCAGCAA
**pLVT7**	NC	TTCTCCGAACGTGTCACGT

### Downregulation of RABEX-5 inhibits colony formation and breast cancer cell proliferation

A CCK-8 assay was used to further explore the ability of RABEX-5 to modulate breast cancer cell proliferation. The MCF-7/KD group displayed significantly decreased proliferation at 24, 48, 72 and 96 h after incubation compared with the MCF-7/NC group (P<0.05, Figure 2C). Meanwhile, the colony formation assay further revealed the effects of RABEX-5 knockdown on the growth of MCF-7 cells. Downregulation of RABEX-5 markedly suppressed the colony formation ability of MCF-7 cells. The MCF-7/KD group had reduced positive colony formation than the MCF-7/NC group (P<0.05, Figure 2D). These data suggest that downregulation of RABEX-5 suppresses breast cancer cell proliferation.

### Downregulation of RABEX-5 inhibits the migration of breast cancer cells

**Figure 3:**
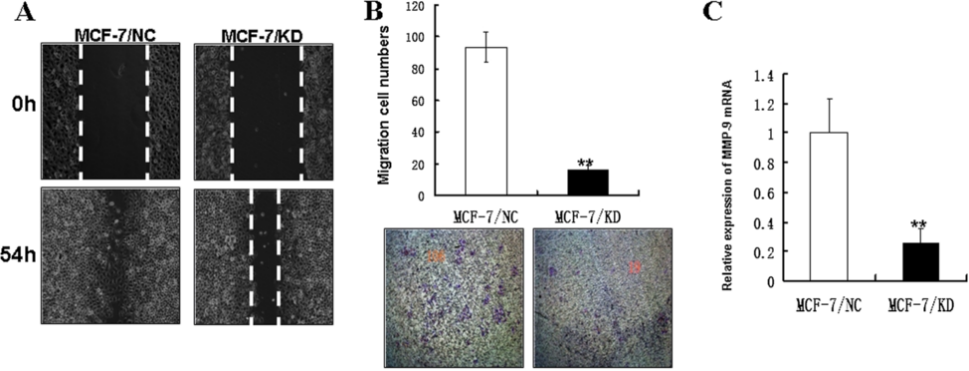
**Downregulation of RABEX-5 expression inhibits cell migration. (A)**, Wound healing assay with MCF-7/NC and MCF-7/KD cells. Microscopic observations were recorded 0 and 54 hours after scratching the cell surface. a representative image from three independent experiments is shown. **(B)**, Transwell assay. Photographs represented the cells travelled through the micropore membrane and histogram showed the percentage of migrant cells. **(C)**, MMP-9 mRNA expression was evaluated by real time-PCR. The asterisk indicates statistical significant difference(P<0.05). Original magnification, ×40.

To investigate the role of RABEX-5 in breast cancer metastasis, we investigated the migratory and invasive capacity of MCF-7/KD and MCF-7/NC cells. To test whether downregulation of RABEX-5 could inhibit tumor cell migration, a wound healing assay was performed. The migration of MCF-7/KD cells across the wound edges was remarkably slower than that of the MCF-7/NC cells at 54 h (Figure 3A). From the transwell assay, we found that the percentage of MCF-7/KD cells that migrated through the micropore membrane was significantly less than the MCF-7/NC cells (P<0.05, Figure 3B). These results indicate that the downregulation of RABEX-5 inhibits the migration of breast cancer cells and that RABEX-5 indeed possesses the ability to promote tumor metastasis and can function as an oncogene in breast cancer.

### Knockdown of RABEX-5 suppresses the expression of MMP-9

MMP-9 is a matrix metalloproteinase that was previously shown to play a critical role in the tumor microenvironment by enhancing cancer cell motility, angiogenesis and cancer growth [16]. Our data have demonstrated that RABEX-5 can promote the migration and invasion of breast cancer cells; however, it is unknown whether RABEX-5 can modulate MMP-9 expression. Therefore, we next examined the expression level of MMP-9 in MCF-7/KD and MCF-7/NC cells using real-time PCR. The expression of MMP-9 was significantly reduced in MCF-7/KD cells compared with MCF-7/NC cells (P<0.05, Figure 3C). These data suggest that knockdown of RABEX-5 suppresses the metastasis of breast cancer cells through the modulation of MMP-9 transcriptional activity.

### Gene silencing of RABEX-5 inhibits breast cancer growth in vivo

**Figure 4:**
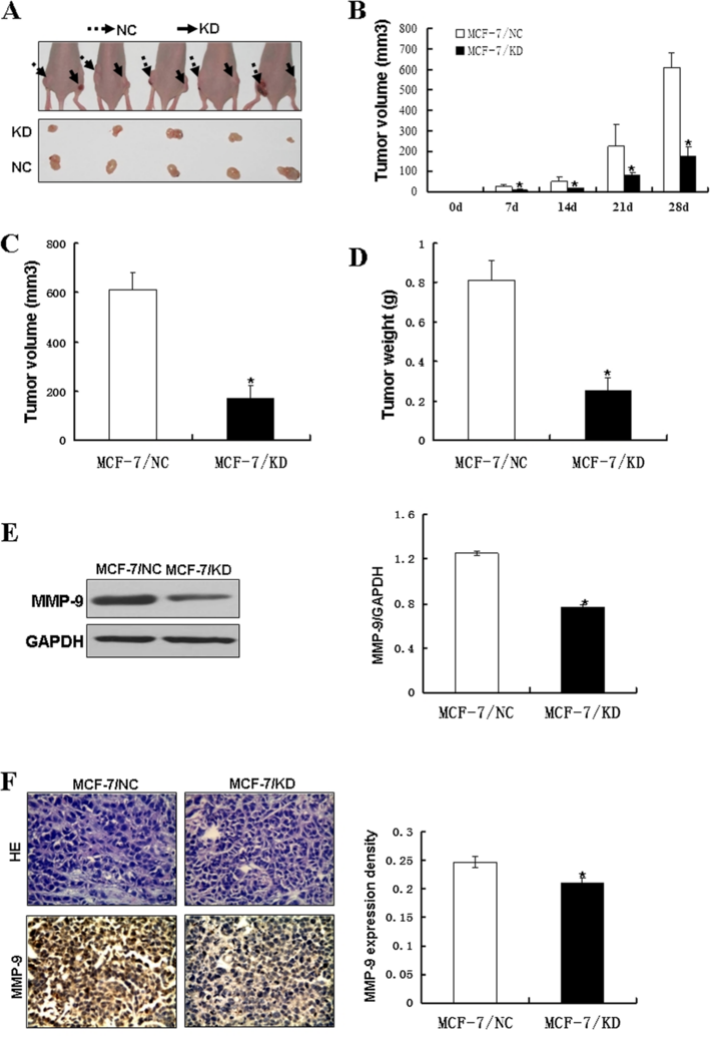
**Gene silencing of RABEX-5 inhibits breast cancer growth in vivo. (A)**, MCF-7/KD cells and MCF-7/NC cells were injected subcutaneously into nude mice. Mice were sacrificed after 4 weeks from transplant. **(B**-**D)**, Tumor volume and tumor weight were measured after dissection. **(B)**, Tumor volume were recorded 0, 7, 14, 21 and 28 days after after tumor cell inoculated, and the final tumor weight **(D)** and volume **(C)** were determined. **(E)**, MMP-9 protein levels in transplantation tumor samples were analyzed by western blot. GAPDH was used as an internal control. **(F)**,The immunohistochemistry analysis of MMP-9 expression in tumors derived from MCF-7/NC group and MCF-7/KD group. Original magnification, ×40. The asterisk indicates statistical significant difference (P<0.05).

Based on the in vitro findings described above, we examined the effect of RABEX-5 silencing on tumor growth in vivo. Xenografts in nude mice were established by subcutaneous injection of MCF-7/KD cells and MCF-7/NC cells into nude mice as described in the Materials and Methods section. Tumor size was monitored every 3 days with a caliper. The tumor growth of the xenografts derived from the MCF-7/NC group was comparable to that of the MCF-7/KD group, showing a marked increase in tumor volume 4 weeks after tumor cell inoculation (P<0.05, Figure 4A, Figure 4B, Figure 4C). In addition, the final mean tumor weight of the MCF-7/KD group was significantly lighter than that of the MCF-7/NC group (P<0.05, Figure 4D), indicating that the silencing of RABEX-5 causes an inhibition of growth of MCF-7 tumors in vivo. Next, western blotting was used to examine the expression of RABEX-5 and MMP-9 in transplantation tumor samples. As shown in Figure 4E, the protein expression level of RABEX-5 and MMP-9 in the MCF-7/KD group was decreased compared with the MCF-7/KD group (P<0.05). Immunohistochemistry was also used to determine the protein expression of RABEX-5 and MMP-9 in the tumor sections. Analysis of RABEX-5 and MMP-9 expression in tumors derived from the MCF-7/NC group indicated strong staining throughout the tumor mass, whereas tumors from the MCF-7/KD group showed less staining only in part of the areas of the tumor mass (Figure 4F). These in vivo data were consistent with the in vitro results and confirmed that the silencing of RABEX-5 inhibits breast cancer growth and progression by modulating MMP-9 transcriptional activity. In summary, RABEX-5 plays an oncogenic role in breast cancer.

## Discussion

RABEX-5 is a guanine nucleotide exchange factor (GEF) for RAB-5 [13], a small GTPase that regulates early endosome fusion and endocytosis [17-21]. RABEX-5 was identified as an interactor of Rabaptin-5 and was found to possess GEF activity toward RAB-5 and related GTPases; both Rabaptin-5 and RABEX-5 are essential for RAB-5-driven endosome fusion. Previous studies have reported that RABEX-5 can specifically bind to the active form of RAB-5, thereby regulating the docking and fusion of endosomal membranes, the motility of endosomes and intracellular signal transduction [22]. It has been demonstrated that the expression of RAB-5 proteins was associated with the development of various malignant tumors of the breast, ovary, and lung [23-25]. However, previous studies have not yet investigated the association between RABEX-5 expression and cancer. In the present study, we demonstrated that RABEX-5 was overexpressed in breast cancer tissues and breast cancer cells; in addition, the influence of RABEX-5 on the biological behavior of breast cancer cells in vitro and in vivo was investigated. Our results argue that RABEX-5 may have an oncogenic effect on breast cancer.

In this study, we found that RABEX-5 was clearly overexpressed in all 5 breast cancer cell lines (MCF-7, MDA-MB-231, T47D, BT549, and SKBR3) and breast cancer tissues that were tested. In contRast, RABEX-5 was expressed at low levels in benign breast tumor tissues and normal breast tissues. The high expression of RABEX-5 in breast cancer cells was consistent with the results obtained from other tumors [14], which indicates that RABEX-5 was involved in tumorigenesis. To explore the importance, role and function of RABEX-5 and to determine how RABEX-5 affects the proliferation, growth, invasion and migration of breast cancer cells, RNAi was used to suppress the expression of RABEX-5. In our studies, four pairs of siRNAs that targeted RABEX-5 and one negative control siRNA were designed. Compared with other gene knockout techniques, this technique is highly efficient, specific, stable, transmissible and hereditable; therefore, it plays an important role in gene function research and gene therapy of tumors [26]. Thus, a lentiviral vector for RNA interference (RNAi) of the RABEX-5 gene was constructed to silence the expression of RABEX-5 in MCF-7 cells.

Real-time PCR and western blots confirmed that the expression of RABEX-5 was suppressed in MCF-7/KD cells. In addition, the colony formation assay and CCK-8 assay demonstrated that the silencing of RABEX-5 altered the proliferation and growth of the cells. After the transfection of RABEX-5 siRNA into MCF-7 cells, the invasion and migration capacities of the cells were significantly altered, as shown by transwell cell invasion and wound healing assays. To further investigate the role of RABEX-5 in tumorigenesis, we established transplanted tumor models in mice, and the results were consisted with our in vitro results. These data suggest a potential role for RABEX-5 in the onset of carcinogenesis in breast cancer. We also studied the expression of RABEX-5 in 60 cases of breast cancer patients and found that RABEX-5 expression was related to axillary lymph node metastasis, which further demonstrated that RABEX-5 played an important role in breast cancer metastasis. In this study, we showed that RABEX-5 potentially acts as a poor prognostic factor for breast cancer because it is associated with the onset of breast cancer and increased metastasis. Thus, it might become a promising therapeutic target for breast cancer.

RABEX-5 inhibition resulted in decreased proliferation and metastasis of breast cancer cells. However, the mechanism remains unclear. MMP-9 is one of the most important members of the MMPs (matrix metalloproteinases). It is produced predominantly by leukocytes and has been extensively studied in cancer and other diseases [27]. MMP-9 is required for physiological processes such as ECM remodeling during growth and development, inflammation, wound healing, angiogenesis, and leukocyte mobilization. It is also involved in pathological processes such as cancer, inflammation, and neural and vascular degenerative diseases [16,28,29]. Early research showed that MMP-9 had a distinct role in tumor angiogenesis, mainly through its ability to regulate the bioavailability of vascular endothelial growth factor (VEGF) [30]. Furthermore, MMP-9 was previously shown to play a critical role in maintaining the tumor microenvironment, leading to enhanced cancer cell motility and cancer growth [16]. In this study, we showed that RABEX-5 silencing triggered a decrease in MMP-9 activation. Therefore, we hypothesize that RABEX-5 promotes the migration and invasion of breast cancer cells through activation of MMP-9.

In conclusion, we found that RABEX-5 expression in breast cancer tissues was significantly higher than in normal breast tissues and benign breast tumor tissues, and it correlated with the clinical feature of axillary lymph node metastasis. Moreover, overexpression of RABEX-5 promotes tumor growth, migration and invasion of breast cancer cells in vitro and in transplanted tumor models. RABEX-5 plays an important oncogenic role in breast cancer. It may also serve as a useful indicator for tumor progression and metastasis, and its effects might be partially mediated by modulation of MMP-9 activation.

## Abbreviations

RABEX-5: A guanine nucleotide exchange factor (GEF) for RAB-5; RNAi: RNA interference; MMP-9: Matrix metallopeptidase 9; VEGF: Vascular endothelial growth factor; PCR: Polymerase chain reaction; CCK-8: A cell count kit-8; IHC: Immunohistochemistry.

## Competing interests

The authors declare that they have no competing interests.

## Authors’ contributions

XZ and JM participated in the study design, constructed lentiviral plasmid vector ,conducted the real-time PCR assays and drafted the manuscript; YJW and YL statisticsed the patient information and conducted immunohistochemical staining; HZL carried out the western bolt assay; QL and XJL carried out the proliferation and cell migration assay; PM conduced the trials in vivo. ; HYL conceived of the study, and participated in its design and coordination, and reviewed the manuscript. All authors read and approved the final manuscript.
